# Supplementary material for: Generation of T cells with reduced off-target cross-reactivities by engineering co-signalling receptors
Source: Nat Biomed Eng. 2026 Jan 2;10(4):753–64. doi: 10.1038/s41551-025-01563-w (PMC7618719; doi:10.1038/s41551-025-01563-w)
Supplement: Supplementary file 1 — Supplementary Figs. 1–5 and Tables 1–4. [file 41551_2025_1563_MOESM1_ESM.pdf]

nature biomedical engineering

Supplementary information

https://doi.org/10.1038/s41551-025-01563-w

# Generation of T cells with reduced off-target cross-reactivities by engineering co-signalling receptors

In the format provided by the authors and unedited

# Supplementary Information:
# Generation of T cells with reduced off-target cross-reactivities by engineering co-signalling receptors

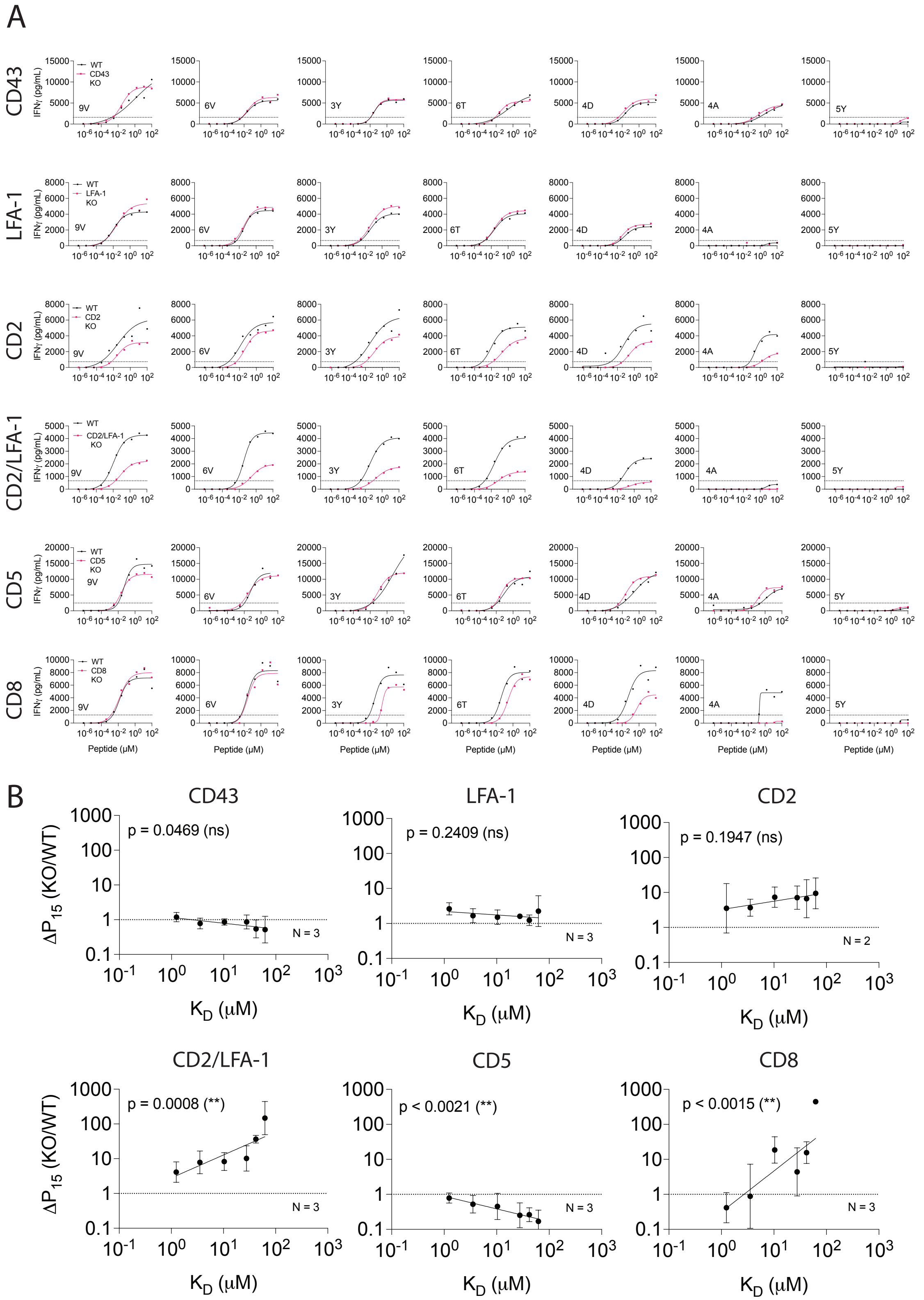


Supplementary Figure 1: **The impact of different T cell co-signalling receptors on ligand sensitivity and discrimination using the secreted cytokine IFN $\gamma$.** **(A)** Representative dose-response and **(B)** Change in potency over affinity as described in Fig. 1D for target killing. Data in (A) are representative of at least N=2 independent experiments with different blood donors. Dashed line in (B) indicates fold change of 1. Data is shown as means ± SDs. Significance of non-zero slope was assessed by a two-tailed F-test.

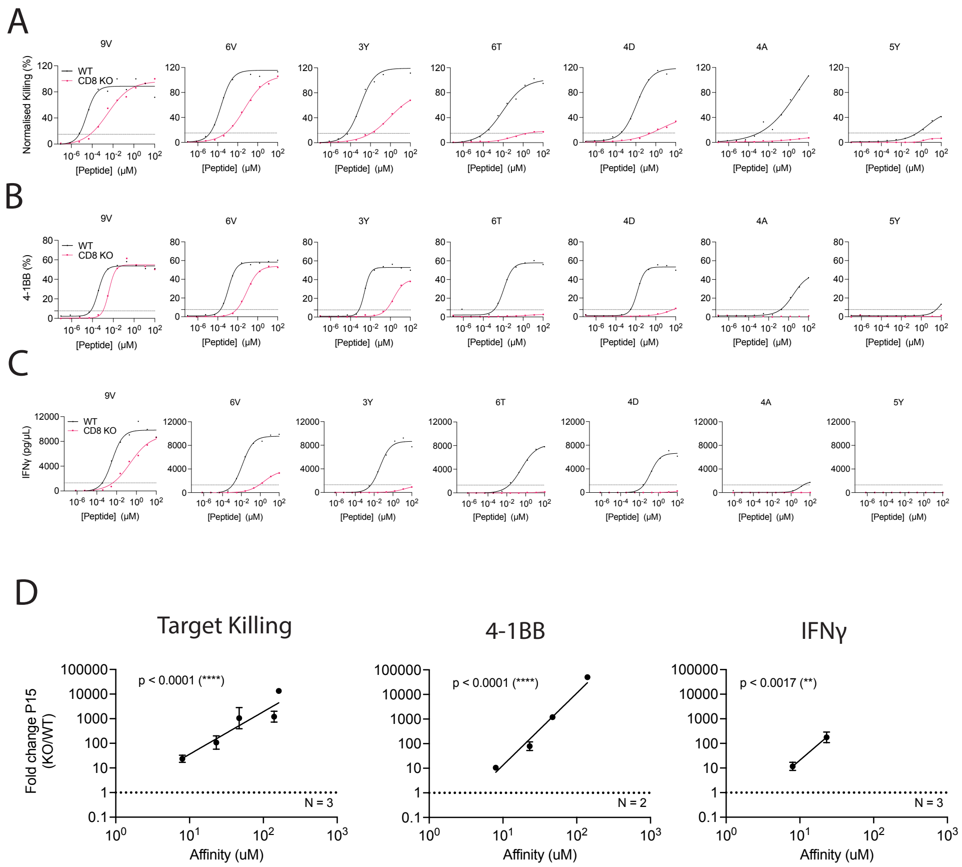


Supplementary Figure 2: **CD8 KO increases the ligand discrimination of the 1G4 TCR.**
**(A to C)** U87 cells were titrated with each of the 7 NY-ESO-1 peptides to stimulate WT or CD8 KO 1G4 TCR-T cells. **(A)** 4-1BB expression was measured after 20 hours. **(B)** Killing of the target U87 cells was measured after 20 hours. **(C)** IFN $\gamma$ secretion was measured after 20 hours. **(D)** Fold change in potency (P15) between KO and WT 1G4 TCR-T cells plotted over TCR/pMHC affinity ($K_D$) (24). Dashed line indicates fold change of 1. Data is shown as means ± SDs. Data in (A), (B) and (C) are representative of at least N=2 independent experiments with different blood donors. Significance of non-zero slope was assessed by a two-tailed F-test.

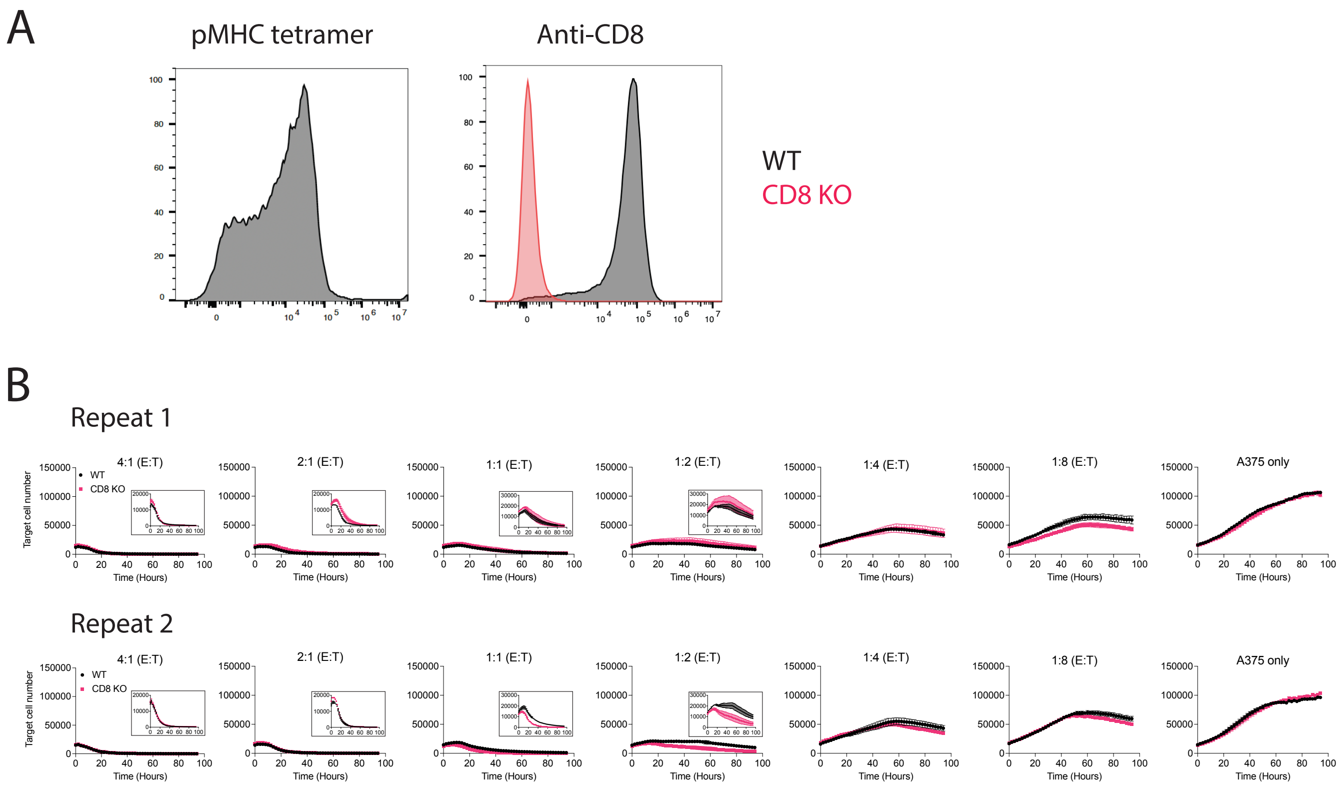


Supplementary Figure 3: **CD8 co-receptor KO abolishes MAGE-A3 TCR cross-reactivity to the self-antigen Titin without reducing responses against MAGE-A3.**

**(A)** Flow cytometry analysis of TCR expression using an EVDPIGHLY HLA-A*01:01 tetramer and of CD8 expression in a3a TCR-T WT or CD8 KO cells. **(B)** A375 cells endogenously expressing the MAGE-A3 protein were co-cultured with WT or CD8 KO cytotoxic a3a TCR-T cells. A375 cell number was measured every two hours. Data in (A) is representative from at least N=3 independent experiments with different blood donors. Data in (B) is shown as means ± SDs of technical triplicates from two independent experiments with different blood donors.

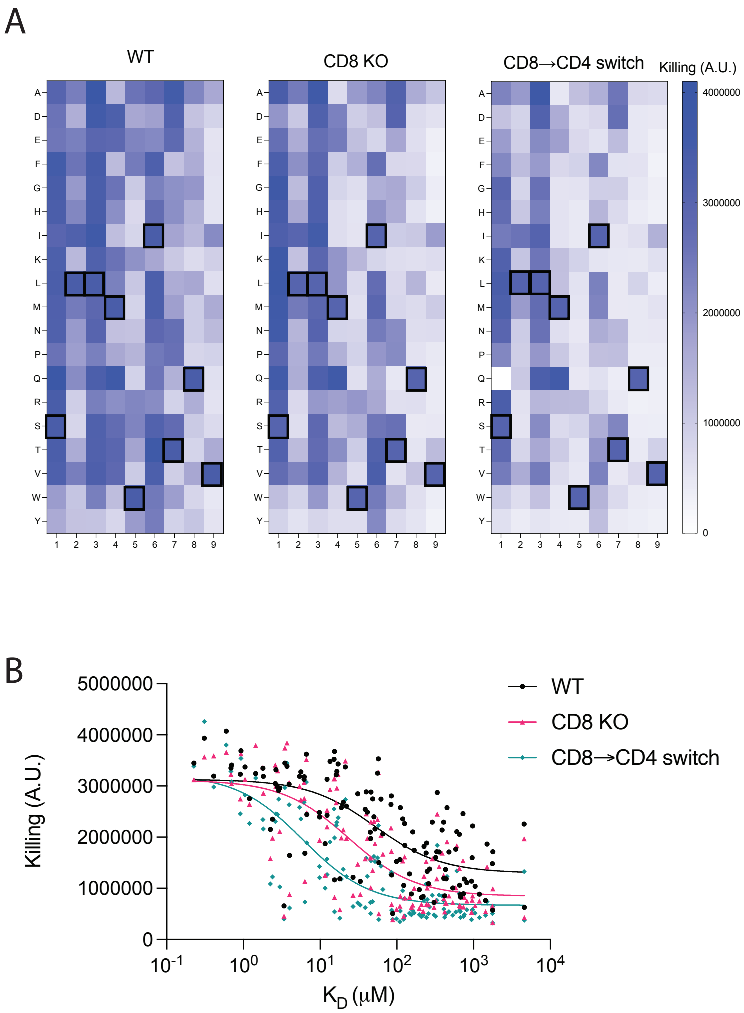


Supplementary Figure 4: **Expression of the incompatible CD4 co-receptor in cytotoxic T cells enhances ligand discrimination against a positional scanning library (1 µM).**

**(A)** U87 cells were individually loaded with 1 µM of each of the 163 peptides in the positional library and co-cultured with T cells. Target killing was measured after 20 hours. Boxed amino acids represent the NY-ESO-1 9V peptide SLLMWITQV. **(B)** Target cell killing from (A) plotted over the TCR/pMHC $K_D$. Data in (A) and (B) are representative data from N=3 independent experiments with different blood donors.

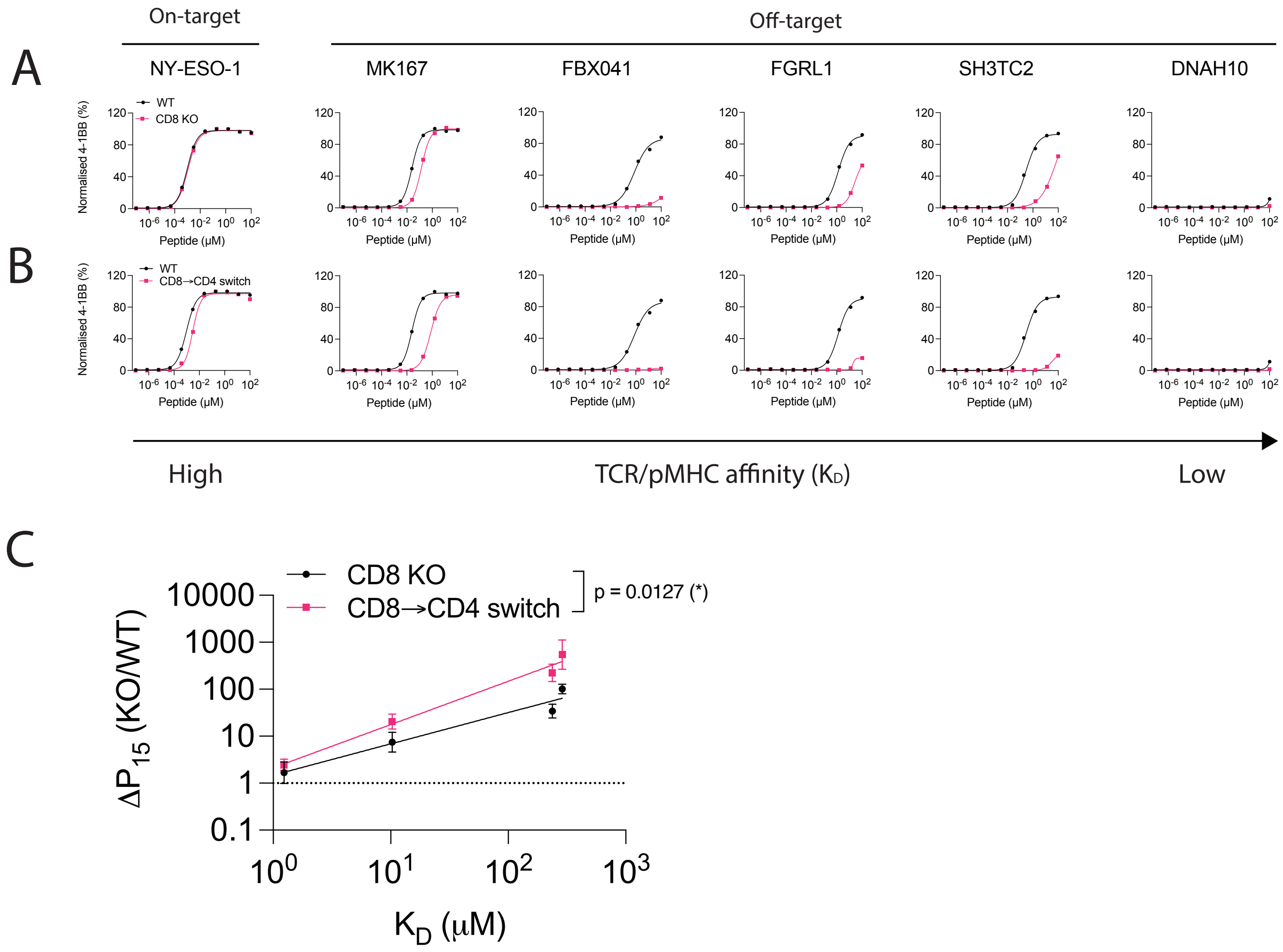


Supplementary Figure 5: **CD8→CD4 co-receptor switch cytotoxic display reduced activation against predicted self-peptides (4-1BB).** **(A)** U87 cells were titrated with each of the predicted self-peptides to stimulate WT or CD8 KO cytotoxic c259 TCR- T cells. 4-1BB expression was measured after 20 hours. **(B)** U87 cells were titrated with each of the predicted self-peptides to stimulate WT or CD8→CD4 co-receptor switch cytotoxic c259 TCR-T cells. 4-1BB expression was measured after 20 hours. **(C)** Fold change in potency (P15) between modified or WT T cells from (A and B) is plotted over TCR/pMHC affinity ($K_D$). Data is shown as means ± SDs. Data in (A) and (B) are representative of N=3 independent experiments with different blood donors. P values were determined by a two-tailed F-test. $*p<0.05$.

## Supplementary Tables

Supplementary Table 1: The c259 TCR affinities to the NY-ESO-1 peptide variants.

| Abbr | Sequence | Mean | SD | N |
|---|---|---|---|---|
| 9V | SLLMWITQV | 1.240 | 0.129 | 3 |
| 6V | SLLMWVTQV | 3.538 | 0.283 | 2 |
| 3Y | SLYMWITQV | 10.350 | 1.634 | 4 |
| 6T | SLLMWTTQV | 27.628 | 4.598 | 11 |
| 4D | SLLDWITQV | 41.777 | 4.703 | 7 |
| 4A | SLLAWITQV | 62.319 | 10.313 | 8 |
| 5Y | SLLMYITQV | 157.666 | 23.818 | 3 |

Supplementary Table 2: **The c259 TCR affinities to the positional scanning peptide library.** Geometric mean, geometric standard deviation across N experiments is reported. $K_D$ values have been excluded if pMHC was unstable (indicated as N/A) or no TCR binding response was observed (indicated as NB).

| **Position 1** | | | | | **Position 2** | | | | | **Position 3** | | | | |
|---|---|---|---|---|---|---|---|---|---|---|---|---|---|---|
| Abbr | Sequence | Geo. mean | Geo. SD | N | Abbr | Sequence | Geo. mean | Geo. SD | N | Abbr | Sequence | Geo. mean | Geo. SD | N |
| **1A** | ALLMWITQV | 2.606 | 2.781 | 2 | **2A** | SALMWITQV | N/A | | | **3A** | SLAMWITQV | 0.596 | 1.057 | 2 |
| **1D** | DLLMWITQV | 18.846 | 1 | 1 | **2D** | SDLMWITQV | N/A | | | **3D** | SLDMWITQV | N/A | | |
| **1E** | ELLMWITQV | N/A | | | **2E** | SELMWITQV | N/A | | | **3E** | SLEMWITQV | N/A | | |
| **1F** | FLLMWITQV | 57.473 | 5.281 | 2 | **2F** | SFLMWITQV | N/A | | | **3F** | SLFMWITQV | 13.441 | 3.048 | 3 |
| **1G** | GLLMWITQV | 3.478 | 3.361 | 3 | **2G** | SGLMWITQV | N/A | | | **3G** | SLGMWITQV | N/A | | |
| **1H** | HLLMWITQV | 21.339 | 10.308 | 3 | **2H** | SHLMWITQV | N/A | | | **3H** | SLHMWITQV | 17.216 | 18.519 | 2 |
| **1I** | ILLMWITQV | 2.787 | 6.294 | 3 | **2I** | SILMWITQV | 5.397 | 63.1 | 2 | **3I** | SLIMWITQV | 6.639 | 11.558 | 3 |
| **1K** | KLLMWITQV | 1.444 | 8.647 | 3 | **2K** | SKLMWITQV | N/A | | | **3K** | SLKMWITQV | N/A | | |
| **1L** | LLLMWITQV | 3.7 | 9.695 | 3 | **2L** | SLLMWITQV | 0.701 | 1.966 | 3 | **3L** | SLLMWITQV | 0.701 | 1.966 | 3 |
| **1M** | MLLMWITQV | N/A | | | **2M** | SMLMWITQV | 6.684 | 23.015 | 2 | **3M** | SLMMWITQV | 2.885 | 3.632 | 3 |
| **1N** | NLLMWITQV | 0.409 | 1 | 1 | **2N** | SNLMWITQV | N/A | | | **3N** | SLNMWITQV | 3.951 | 19.691 | 2 |
| **1P** | PLLMWITQV | N/A | | | **2P** | SPLMWITQV | N/A | | | **3P** | SLPMWITQV | N/A | | |
| **1Q** | QLLMWITQV | 0.309 | 1.403 | 2 | **2Q** | SQLMWITQV | 2.241 | 14.094 | 2 | **3Q** | SLQMWITQV | 0.688 | 6.074 | 2 |
| **1R** | RLLMWITQV | 0.226 | 1.082 | 2 | **2R** | SRLMWITQV | N/A | | | **3R** | SLRMWITQV | N/A | | |
| **1S** | SLLMWITQV | 0.701 | 1.966 | 3 | **2S** | SSLMWITQV | N/A | | | **3S** | SLSMWITQV | 1.82 | 2.026 | 3 |
| **1T** | TLLMWITQV | 1.046 | 3.568 | 2 | **2T** | STLMWITQV | N/A | | | **3T** | SLTMWITQV | 6.228 | 1 | 1 |
| **1V** | VLLMWITQV | 0.91 | 1.746 | 3 | **2V** | SVLMWITQV | 50.287 | 1 | 1 | **3V** | SLVMWITQV | 2.657 | 1 | 1 |
| **1W** | WLLMWITQV | 11.631 | 1.329 | 3 | **2W** | SWLMWITQV | 33.447 | 3.481 | 3 | **3W** | SLWMWITQV | 9.861 | 1.691 | 3 |
| **1Y** | YLLMWITQV | 3.993 | 1.292 | 3 | **2Y** | SYLMWITQV | 18.095 | 1.288 | 3 | **3Y** | SLYMWITQV | 15.39 | 1.34 | 3 |
| **Position 4** | | | | | **Position 5** | | | | | **Position 6** | | | | |
| Abbr | Sequence | Geo. mean | Geo. SD | N | Abbr | Sequence | Geo. mean | Geo. SD | N | Abbr | Sequence | Geo. mean | Geo. SD | N |
| **4A** | SLLAWITQV | 431.199 | 19.596 | 2 | **5A** | SLLMAITQV | 450.833 | 2.219 | 2 | **6A** | SLLMWATQV | 46.1 | 1.912 | 3 |
| **4D** | SLLDWITQV | 55.894 | 4.407 | 3 | **5D** | SLLMDITQV | 1779.52 | 1 | 1 | **6D** | SLLMWDTQV | 226.549 | 1.541 | 2 |
| **4E** | SLLEWITQV | 335.407 | 3.105 | 3 | **5E** | SLLMEITQV | 406.72 | 2.54 | 3 | **6E** | SLLMWETQV | 971.245 | 1.531 | 2 |
| **4F** | SLLFWITQV | 248.389 | 1.439 | 3 | **5F** | SLLMFITQV | 730.463 | 1.622 | 3 | **6F** | SLLMWFTQV | 16.258 | 4.03 | 3 |
| **4G** | SLLGWITQV | 111.398 | 2.492 | 3 | **5G** | SLLMGITQV | 658.784 | 1.293 | 2 | **6G** | SLLMWGTQV | 118.893 | 1 | 1 |
| **4H** | SLLHWITQV | 161.301 | 1.704 | 3 | **5H** | SLLMHITQV | 1436.123 | 1 | 1 | **6H** | SLLMWHTQV | 61.979 | 1.624 | 3 |
| **4I** | SLLIWITQV | 85.441 | 1.52 | 3 | **5I** | SLLMIITQV | 220.473 | 1 | 1 | **6I** | SLLMWITQV | 0.701 | 1.966 | 3 |
| **4K** | SLLKWITQV | 22.035 | 4.9 | 3 | **5K** | SLLMKITQV | 654.509 | 2.109 | 2 | **6K** | SLLMWKTQV | 476.51 | 1.479 | 3 |
| **4L** | SLLLWITQV | 2.392 | 17.993 | 3 | **5L** | SLLMLITQV | 815.925 | 1.446 | 3 | **6L** | SLLMWLTQV | 5.596 | 5.279 | 3 |
| **4M** | SLLMWITQV | 0.701 | 1.966 | 3 | **5M** | SLLMMITQV | 670.428 | 1.447 | 2 | **6M** | SLLMWMTQV | 16.572 | 16.638 | 3 |
| **4N** | SLLNWITQV | 26.905 | 2.998 | 3 | **5N** | SLLMNITQV | 758.195 | 1 | 1 | **6N** | SLLMWNTQV | 40.021 | 2.134 | 3 |
| **4P** | SLLPWITQV | 241.083 | 2.6 | 3 | **5P** | SLLMPITQV | N/A | | | **6P** | SLLMWPTQV | 41.366 | 1 | 1 |
| **4Q** | SLLQWITQV | 0.927 | 1.821 | 3 | **5Q** | SLLMQITQV | 154.879 | 1 | 1 | **6Q** | SLLMWQTQV | 38.707 | 1.705 | 3 |
| **4R** | SLLRWITQV | 604.902 | 1.555 | 2 | **5R** | SLLMRITQV | 4576.089 | 1 | 1 | **6R** | SLLMWRTQV | 389.248 | 1.019 | 2 |
| **4S** | SLLSWITQV | 48.243 | 1.186 | 3 | **5S** | SLLMSITQV | 289.89 | 1.941 | 3 | **6S** | SLLMWSTQV | 12.323 | 2.107 | 3 |
| **4T** | SLLTWITQV | 2.985 | 1.985 | 3 | **5T** | SLLMTITQV | 584.582 | 1.56 | 3 | **6T** | SLLMWTTQV | 15.427 | 1.101 | 3 |
| **4V** | SLLVWITQV | 30.833 | 1.08 | 2 | **5V** | SLLMVITQV | 1048.028 | 1.686 | 2 | **6V** | SLLMWVTQV | 1.796 | 1.671 | 3 |
| **4W** | SLLWWITQV | 146.053 | 1.285 | 3 | **5W** | SLLMWITQV | 0.701 | 1.966 | 3 | **6W** | SLLMWWTQV | 9.808 | 1.354 | 3 |
| **4Y** | SLLYWITQV | 208.894 | 1.518 | 3 | **5Y** | SLLMYITQV | 243.945 | 1.92 | 3 | **6Y** | SLLMWYTQV | 12.347 | 1.439 | 3 |
| **Position 7** | | | | | **Position 8** | | | | | **Position 9** | | | | |
| Abbr | Sequence | Geo. mean | Geo. SD | N | Abbr | Sequence | Geo. mean | Geo. SD | N | Abbr | Sequence | Geo. mean | Geo. SD | N |
| **7A** | SLLMWIAQV | 15.201 | 3.121 | 3 | **8A** | SLLMWITAV | 126.651 | 3.221 | 3 | **9A** | SLLMWITQA | 6.31 | 18.975 | 3 |
| **7D** | SLLMWIDQV | 6.163 | 9.833 | 3 | **8D** | SLLMWITDV | 440.519 | 1.688 | 2 | **9D** | SLLMWITQD | N/A | | |
| **7E** | SLLMWIEQV | 85.179 | 1.266 | 3 | **8E** | SLLMWITEV | 1758.587 | 2.616 | 3 | **9E** | SLLMWITQE | N/A | | |
| **7F** | SLLMWIFQV | 408.909 | 1.802 | 3 | **8F** | SLLMWITFV | 961.039 | 1 | 1 | **9F** | SLLMWITQF | 1773.142 | 1 | 1 |
| **7G** | SLLMWIGQV | 65.685 | 9.19 | 2 | **8G** | SLLMWITGV | 45.631 | 3.139 | 3 | **9G** | SLLMWITQG | N/A | | |
| **7H** | SLLMWIHQV | 45.044 | 4.503 | 3 | **8H** | SLLMWITHV | 99.112 | 1.372 | 3 | **9H** | SLLMWITQH | N/A | | |
| **7I** | SLLMWIIQV | 168.64 | 2.155 | 3 | **8I** | SLLMWITIV | 267.092 | 1.481 | 2 | **9I** | SLLMWITQI | 51.817 | 1 | 1 |
| **7K** | SLLMWIKQV | 259.139 | 1 | 1 | **8K** | SLLMWITKV | 305.156 | 1.099 | 2 | **9K** | SLLMWITQK | N/A | | |
| **7L** | SLLMWILQV | 321.614 | 1.129 | 2 | **8L** | SLLMWITLV | 357.078 | 2.024 | 2 | **9L** | SLLMWITQL | 3.39 | 1.913 | 2 |
| **7M** | SLLMWIMQV | 127.392 | 2.346 | 3 | **8M** | SLLMWITMV | 536.124 | 1.767 | 3 | **9M** | SLLMWITQM | N/A | | |
| **7N** | SLLMWINQV | 20.053 | 7.134 | 3 | **8N** | SLLMWITNV | N/A | | | **9N** | SLLMWITQN | N/A | | |
| **7P** | SLLMWIPQV | 83.799 | 4.303 | 3 | **8P** | SLLMWITPV | N/A | | | **9P** | SLLMWITQP | N/A | | |
| **7Q** | SLLMWIQQV | 58.27 | 1.297 | 3 | **8P** | SLLMWITQV | 0.701 | 1.966 | 3 | **9Q** | SLLMWITQQ | 4607.455 | 1 | 1 |
| **7R** | SLLMWIRQV | 738.23 | 1.616 | 2 | **8R** | SLLMWITRV | 109.572 | 1.42 | 3 | **9R** | SLLMWITQR | N/A | | |
| **7S** | SLLMWISQV | 1.199 | 1.839 | 3 | **8S** | SLLMWITSV | 122.849 | 1.544 | 3 | **9S** | SLLMWITQS | N/A | | |
| **7T** | SLLMWITQV | 0.701 | 1.966 | 3 | **8T** | SLLMWITTV | 420.7 | 2.238 | 3 | **9T** | SLLMWITQT | N/A | | |
| **7V** | SLLMWIVQV | 196.983 | 2.279 | 3 | **8V** | SLLMWITVV | 330.714 | 5.072 | 2 | **9V** | SLLMWITQV | 0.701 | 1.966 | 3 |
| **7W** | SLLMWIWQV | 1497.217 | 4.298 | 2 | **8W** | SLLMWITWV | 677.351 | 3.87 | 3 | **9W** | SLLMWITQW | 88.229 | 1 | 1 |
| **7Y** | SLLMWIYQV | 1190.897 | 2.148 | 3 | **8Y** | SLLMWITYV | 939.924 | 3.947 | 2 | **9Y** | SLLMWITQY | N/A | | |

Supplementary Table 3: The c259 TCR affinities to predicted self-peptides.

| Gene | Sequence | Mean | SD | N |
|---|---|---|---|---|
| NY-ESO-1 (9V) | SLLMWITQV | 1.240 | 0.129 | 3 |
| MKI67 | FLTLWLTQV | 10.314 | 3.612 | 5 |
| FBX041 | MLAQWCTQA | 141.35 | 60.426 | 4 |
| FGFRL1 | TLLLWLCQA | 237.775 | 116.323 | 4 |
| SH3TC2 | QVFLWLAQV | 288.480 | 117.831 | 5 |
| DNAH10 | CVINWLNQI | 412.425 | 225.050 | 4 |

Supplementary Table 4: sgRNA sequences.

| Target gene | sgRNA sequence |
|---|---|
| CD8α | ATACTGTTGTGCGCACATCG<br>GTTAGACGTATCTCGCCGAA<br>GCTGCTGTCCAACCCGACGT<br>GAGCAAGGCGGTCACTGGTA |
| CD5 | GCAGACTTTTGACGCTTGAC<br>CCGTTCCAACTCGAAGTGCC<br>ATCATCTGCTACGGACAACT<br>AGGTCTACCTCAAGGACGGA |
| CD43 | GGCTCGCTAGTAGAGACCAA<br>GCACCAATGGAAGTCCAAAG<br>AGGTTGTTGGCTCAGGTAAA |
| CD2 | CAAGGCACCCCAGGTTTCCA<br>CAAAGAGATTACGAATGCCT<br>CTTGTAGATATCCTGATCAT<br>GCATCTGAAGACCGATGATC |
| CD11a | CTTTGGATACCGCGTCCTGC<br>CAAGTACTTGGAGGTATAGT<br>GTAACACAGGCCACTCAGAT<br>GUAGCUCGAGGCCGGCGCUG |
| CD4 | GTCAGCGCGATCATTCAGCT<br>GAGGTGCAATTGCTAGTGTT<br>AACTGTAAAGGCGAGTGGGA<br>CTGTTTTCGCTTCAAGGGCC |
| TRAC | AGAGTCTCTCAGCTGGTACA<br>TGTGCTAGACATGAGGTCTA |
| TRBC | GCAGTATCTGGAGTCATTGA<br>GGAGAATGACGAGTGGACCC |
