# Supplementary material for: Generation of T cells with reduced off-target cross-reactivities by engineering co-signalling receptors
Source: Nat Biomed Eng. 2026 Jan 2;10(4):753–64. doi: 10.1038/s41551-025-01563-w (PMC7618719; doi:10.1038/s41551-025-01563-w)
Supplement: Supplementary file 2 — Reporting Summary [file 41551_2025_1563_MOESM2_ESM.pdf]

nature portfolio

Corresponding author(s): Omer Dushek

Last updated by author(s): 12-09-2024

Reporting Summary

Nature Portfolio wishes to improve the reproducibility of the work that we publish. This form provides structure for consistency and transparency in reporting. For further information on Nature Portfolio policies, see our Editorial Policies and the Editorial Policy Checklist.

Statistics

For all statistical analyses, confirm that the following items are present in the figure legend, table legend, main text, or Methods section.

n/a	Confirmed	
☐	☒	The exact sample size (*n*) for each experimental group/condition, given as a discrete number and unit of measurement
☐	☒	A statement on whether measurements were taken from distinct samples or whether the same sample was measured repeatedly
☐	☒	The statistical test(s) used AND whether they are one- or two-sided<br>*Only common tests should be described solely by name; describe more complex techniques in the Methods section.*
☐	☒	A description of all covariates tested
☐	☒	A description of any assumptions or corrections, such as tests of normality and adjustment for multiple comparisons
☐	☒	A full description of the statistical parameters including central tendency (e.g. means) or other basic estimates (e.g. regression coefficient) AND variation (e.g. standard deviation) or associated estimates of uncertainty (e.g. confidence intervals)
☐	☒	For null hypothesis testing, the test statistic (e.g. *F*, *t*, *r*) with confidence intervals, effect sizes, degrees of freedom and *P* value noted<br>*Give P values as exact values whenever suitable.*
☒	☐	For Bayesian analysis, information on the choice of priors and Markov chain Monte Carlo settings
☒	☐	For hierarchical and complex designs, identification of the appropriate level for tests and full reporting of outcomes
☒	☐	Estimates of effect sizes (e.g. Cohen's *d*, Pearson's *r*), indicating how they were calculated

Our web collection on statistics for biologists contains articles on many of the points above.

Software and code

Policy information about availability of computer code

Data collection	No software was used for data collection.
Data analysis	FlowJo v10.10.0 (BD Biosciences) and GraphPad Prism v10.2.1 (GraphPad Software).

For manuscripts utilizing custom algorithms or software that are central to the research but not yet described in published literature, software must be made available to editors and reviewers. We strongly encourage code deposition in a community repository (e.g. GitHub). See the Nature Portfolio guidelines for submitting code & software for further information.

Data

Policy information about availability of data

All manuscripts must include a data availability statement. This statement should provide the following information, where applicable:

- Accession codes, unique identifiers, or web links for publicly available datasets
- A description of any restrictions on data availability
- For clinical datasets or third party data, please ensure that the statement adheres to our policy

All data is included within the manuscript and supplementary information. Source data is provided directly with the manuscript.

Research involving human participants, their data, or biological material

Policy information about studies with human participants or human data. See also policy information about sex, gender (identity/presentation), and sexual orientation and race, ethnicity and racism.

Reporting on sex and gender	This information has not been collected from the participants.
Reporting on race, ethnicity, or other socially relevant groupings	This information has not been collected from the participants.
Population characteristics	This information has not been collected from the participants.
Recruitment	T cells were isolated from anonymised leukocyte cones purchased from the NHS Blood Donor Centre at the John Radcliffe Hospital (Oxford University Hospitals). As a result of the anonymised nature of the cones, biological sex and gender were not variables in the present study and were therefore randomised, and as a result the authors were blinded to these variables.
Ethics oversight	Ethical approval was provided by the Medical Sciences Inter-divisional Research Ethics Committee (IDREC) at the University of Oxford (R51997/RE001).

Note that full information on the approval of the study protocol must also be provided in the manuscript.

Field-specific reporting

Please select the one below that is the best fit for your research. If you are not sure, read the appropriate sections before making your selection.

☒ Life sciences ☐ Behavioural & social sciences ☐ Ecological, evolutionary & environmental sciences

For a reference copy of the document with all sections, see nature.com/documents/nr-reporting-summary-flat.pdf

Life sciences study design

All studies must disclose on these points even when the disclosure is negative.

Sample size	Sample sizes were determined on the basis of similar published studies and of preliminary experiments.
Data exclusions	No data was excluded.
Replication	All attempts at replication were successful.
Randomization	No randomization was applied.
Blinding	No blinding was applied.

Reporting for specific materials, systems and methods

We require information from authors about some types of materials, experimental systems and methods used in many studies. Here, indicate whether each material, system or method listed is relevant to your study. If you are not sure if a list item applies to your research, read the appropriate section before selecting a response.

Materials & experimental systems

n/a	Involved in the study	
☐	☒	Antibodies
☐	☒	Eukaryotic cell lines
☒	☐	Palaeontology and archaeology
☒	☐	Animals and other organisms
☒	☐	Clinical data
☒	☐	Dual use research of concern
☒	☐	Plants

Methods

n/a	Involved in the study	
☒	☐	ChIP-seq
☐	☒	Flow cytometry
☒	☐	MRI-based neuroimaging

Antibodies

Antibodies used	CD45: Clone HI30 BV421 Biolegend RRID:AB_2561357;<br>CD3: Clone OKT3 488 Biolegend RRID:AB_571877;

4-1BB: Clone 4B4-1 AF647 Biolegend RRID:AB_2566258;
CD69: FN50 AF647 Biolegend RRID:AB_528871;
CD8alpha: Clone HIT8 PE Biolegend RRID:AB_314112;
CD4: Clone RPA-T4 PE Biolegend RRID:AB_314075;
CD43: Clone CD43-10G7 PE Biolegend RRID:AB_2255209;
CD11alpha: Clone TS2/4 PE Biolegend RRID:AB_10660819;
CD5: Clone UCHT2 PE Biolegend RRID:AB_314094;
CD2: Clone TS1/8 PE Biolegend RRID:AB_314758;
TCR V13.1: Clone H131 APC Biolegend RRID:AB_2728348;

Validation	All antibodies used are commercially available, and were validated by the manufacturer.

Eukaryotic cell lines

Policy information about cell lines and Sex and Gender in Research

Cell line source(s)	U87 cell line (Source: ATCC).<br>T2 cell line (Source: ATCC).<br>Nalm6 cell line (Source: Crystal Mackall Lab).<br>A375 cell line (Source: ATCC).
Authentication	The cell lines were not authenticated.
Mycoplasma contamination	Cell lines tested negative for mycoplasma contamination.
Commonly misidentified lines (See ICLAC register)	No commonly misidentified cell lines were used.

Plants

Seed stocks	*Report on the source of all seed stocks or other plant material used. If applicable, state the seed stock centre and catalogue number. If plant specimens were collected from the field, describe the collection location, date and sampling procedures.*
Novel plant genotypes	*Describe the methods by which all novel plant genotypes were produced. This includes those generated by transgenic approaches, gene editing, chemical/radiation-based mutagenesis and hybridization. For transgenic lines, describe the transformation method, the number of independent lines analyzed and the generation upon which experiments were performed. For gene-edited lines, describe the editor used, the endogenous sequence targeted for editing, the targeting guide RNA sequence (if applicable) and how the editor was applied.*
Authentication	*Describe any authentication procedures for each seed stock used or novel genotype generated. Describe any experiments used to assess the effect of a mutation and, where applicable, how potential secondary effects (e.g. second site T-DNA insertions, mosiacism, off-target gene editing) were examined.*

Flow Cytometry

Plots

Confirm that:

☒ The axis labels state the marker and fluorochrome used (e.g. CD4-FITC).

☒ The axis scales are clearly visible. Include numbers along axes only for bottom left plot of group (a 'group' is an analysis of identical markers).

☒ All plots are contour plots with outliers or pseudocolor plots.

☒ A numerical value for number of cells or percentage (with statistics) is provided.

Methodology

Sample preparation	Cells were stained in staining buffer (PBS, 1% BSA,1:200 diluted antibody) for 20 minutes at 4°C, washed with PBS and analysed.
Instrument	BD LRSFortessa X-20 cell analyzer (BD biosciences) and CytoFLEX flow cytometer (Beckman Coulter)
Software	FlowJo v10.10.0 (BD Biosciences)
Cell population abundance	Following CRISPR/Cas9 knock-out, T cells were purified using a PE-conjugated target-specific antibody and MojoSort anti-PE nanobeads. Following nanobead purification, T cell populations were characterised by staining with the target-specific antibody depending on the knock-out performed.

Gating strategy

The starting cell population was gated on a linear SSC-A/FSC-A plot. Single cells were discriminated on a linear FSC-H/FSC-W plot. In co-culture experiments using U87 cells, T cells were gated as CD45 positive. In co-culture experiments using Nalm6 or T2 cells, T cells were gated as CD3 positive. Positive/negative populations were determined with negative controls.

☐ Tick this box to confirm that a figure exemplifying the gating strategy is provided in the Supplementary Information.
