# Supplementary material for: Generation of T cells with reduced off-target cross-reactivities by engineering co-signalling receptors
Source: Nat Biomed Eng. Author manuscript; Available in PMC 2026 Feb 8. (PMC7618719; doi:10.1038/s41551-025-01563-w)
Supplement: Supplementary file 2 [file EMS212284-supplement-Supplementary_Information.pdf]

https://doi.org/10.1038/s41551-025-01563-w

Supplementary information The online version contains supplementary material available at https://doi.org/10.1038/s41551-025-01563-w.
